# Adjacent intact nociceptive neurons drive the acute outburst of pain following peripheral axotomy

**DOI:** 10.1038/s41598-019-44172-9

**Published:** 2019-05-21

**Authors:** Zhiyong Chen, Tao Wang, Yehong Fang, Dan Luo, Michael Anderson, Qian Huang, Shaoqiu He, Xiaodan Song, Huan Cui, Xinzhong Dong, Yikuan Xie, Yun Guan, Chao Ma

**Affiliations:** 1 Institute of Basic Medical Sciences, Department of Human Anatomy, Histology and Embryology, Neuroscience Center, Chinese Academy of Medical Sciences, School of Basic Medicine, Peking Union Medical College, Beijing, 100005 China; 2 Joint Laboratory of Anesthesia and Pain, Peking Union Medical College, Beijing, 100730 China; 3 Department of Anesthesiology and Critical Care Medicine, Johns Hopkins University, School of Medicine, Baltimore, Maryland 21205 USA; 4 Department of Neurosurgery, Xuanwu Hospital, Capital Medical University, Beijing, 100053 China; 5 National Key Laboratory of Medical Molecular Biology & Department of Immunology, Institute of Basic Medical Sciences, Chinese Academy of Medical Sciences, Beijing, 100005 China; 6 College of Pharmacy, Harbin Medical University, Harbin, 150081 China; 7 The Solomon H. Snyder Department of Neuroscience, Center for Sensory Biology, Johns Hopkins University, School of Medicine, Baltimore, Maryland 21205 USA; 8 Department of Neurological Surgery, Johns Hopkins University, School of Medicine, Baltimore, Maryland 21205 USA; 9 Howard Hughes Medical Institute, Johns Hopkins University, School of Medicine, Baltimore, Maryland 21205 USA

**Keywords:** Neuroscience, Peripheral nervous system

## Abstract

Injury of peripheral nerves may quickly induce severe pain, but the mechanism remains obscure. We observed a rapid onset of spontaneous pain and evoked pain hypersensitivity after acute transection of the L5 spinal nerve (SNT) in awake rats. The outburst of pain was associated with a rapid development of spontaneous activities and hyperexcitability of nociceptive neurons in the adjacent uninjured L4 dorsal root ganglion (DRG), as revealed by both *in vivo* electrophysiological recording and high-throughput calcium imaging *in vivo*. Transection of the L4 dorsal root or intrathecal infusion of aminobutyrate aminotransferase inhibitor attenuated the spontaneous activity, suggesting that retrograde signals from the spinal cord may contribute to the sensitization of L4 DRG neurons after L5 SNT. Electrical stimulation of low-threshold afferents proximal to the axotomized L5 spinal nerve attenuated the spontaneous activities in L4 DRG and pain behavior. These findings suggest that peripheral axotomy may quickly induce hyperexcitability of uninjured nociceptors in the adjacent DRG that drives an outburst of pain.

## Introduction

Denervation from axotomy causes a loss of peripheral sensory inputs. Yet, pain can develop rapidly after nerve injury in patients^1–4^. Neuropathic pain-related manifestations, such as spontaneous pain and tactile and heat hypersensitivity, are also observed in animal models of nerve injury and may last for weeks. Moreover, owing to inherent limitations from anesthesia and acute postoperative pain associated with common surgical procedures, the earliest time to onset of pain after nerve injury has yet to be demonstrated in animal models.

Studies in both rodents and non-human primates showed that spontaneous activity (SA) and sensitization of primary nociceptive neurons may contribute to ongoing pain and evoked pain hypersensitivity after injury^5–9^. Yet, these neurophysiologic changes were thought to occur hours after nerve injury^10–12^. Although it is known that nerve injury can induce immediate and lasting pain, the underlying mechanisms remain obscure. Here, we developed a model of axotomy in awake rats by quickly withdrawing a pre-implanted silk suture surrounding the L5 spinal nerve. This acute L5 spinal nerve transection (SNT) model allowed us to assess changes in animal pain behavior immediately after injury, and facilitated mechanistic studies of early changes in neuron excitability by electrophysiologic recording and high-throughput calcium imaging *in vivo*. Our findings unravel a novel neurophysiologic mechanism that may underlie the early onset of pain after axotomy of peripheral nerves, and suggest the utility of low-intensity electrical nerve stimulation for inhibiting the onset of neuronal sensitization and outburst of pain after peripheral nerve injury.

## Results

### Rapid onset of neuropathic pain-related behavior after acute L5 spinal nerve transection

**Figure 1:**
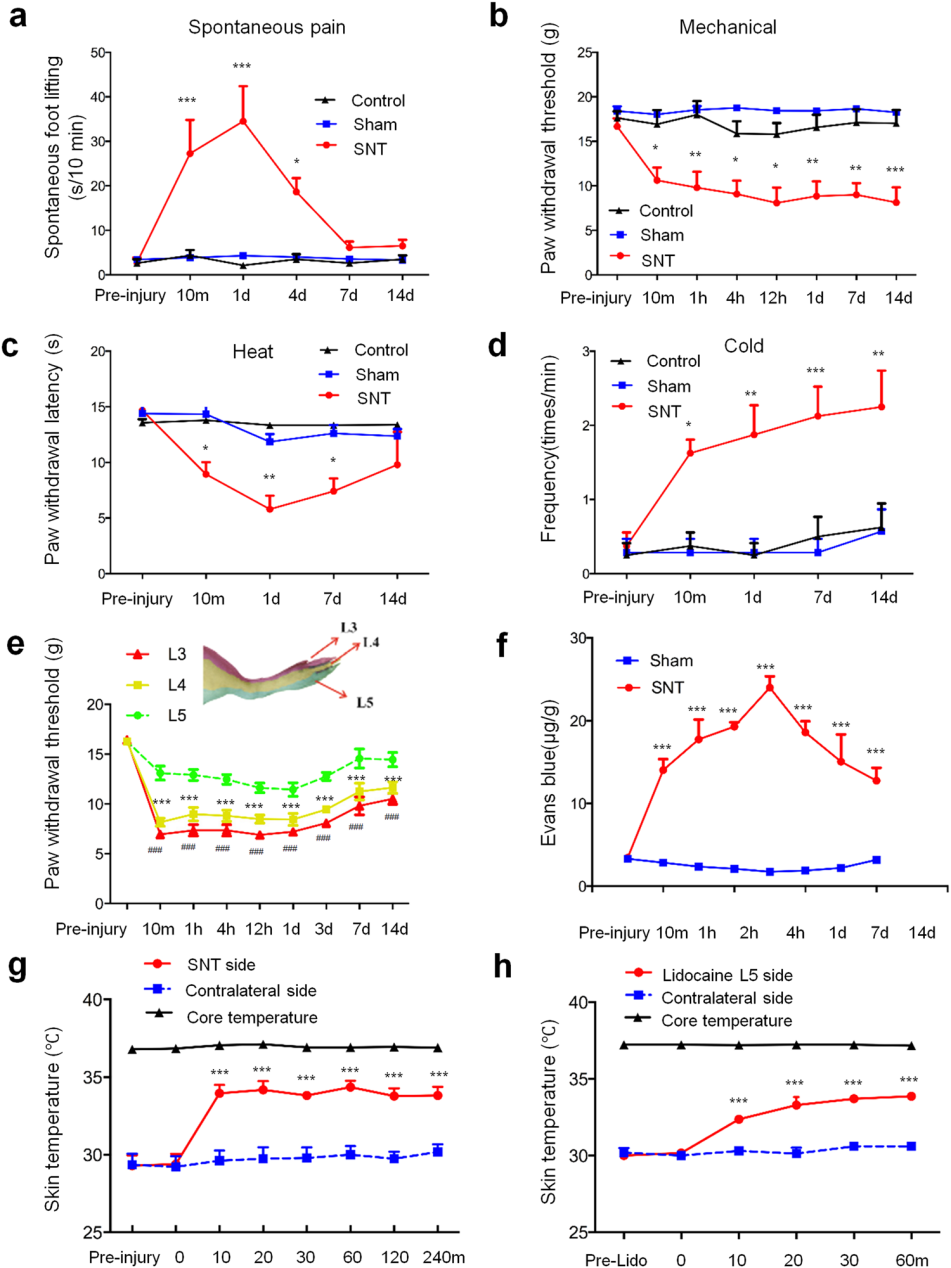
Neuropathic pain-related behavior and neurogenic inflammation after acute L5 SNT in rats. (**a**) Frequency of spontaneous foot lifting at 0 to 14 days after acute L5 SNT (n = 8/group). SNT vs Sham: F_(1, 14)_ = 24.62, ***P = 0.0002; 10 min: *P* < 0.0001; 1 d: *P* < 0.0001; 4 d: *P* = 0.0286. (**b**) PWT of the hind paw ipsilateral to the side of injury from 10 min to 14 days after SNT (n = 8/group). SNT vs Sham: F_(1, 14)_ = 35.41, ***P < 0.0001; 10 min: *P* = 0.0021; 1 h: *P* = 0.0003; 4 h: *P* = 0.0006; 12 h: *P* = 0.0007; 1d: *P* = 0.0007; 7d: *P* = 0.0003; 14d: *P* < 0.0001. (**c**) Changes in the ipsilateral paw withdrawal latency to heat stimuli from 10 min to 14 days after SNT (n = 8/group). SNT vs Sham: F_(1, 14)_ = 9.140, **P = 0.0091; 10 min: *P* = 0.0282; 1d: *P* = 0.0011; 7d: *P* = 0.0196, 14 d: *P* = 0.3120. (**d**) Frequency of licking/biting of the ipsilateral hind paw within 3 min after acetone stimulus (n = 8/group). SNT vs Sham: F_(1, 14)_ = 16.75, **P = 0.0011; 10 min: *P* = 0.0211; 1d: *P* = 0.0035; 7d: *P* = 0.0013; 14 d: *P* = 0.0035. (**e**) The ipsilateral PWTs to stimulation applied to the L3, L4, and L5 dermatomes after L5 SNT (n = 8/Group). Two-way mixed model ANOVA with Tukey’s multiple comparisons test, F_(2, 21)_ = 63.09, *P* < 0.0001. (L5 vs. L4, 10 min: *P* < 0.0001; 1 h: *P* < 0.0001; 4 h: *P* < 0.0001; 12 h: *P* = 0.0002; 1d: *P* = 0.0003; 3d: *P* < 0.0001; 7d: *P* < 0.0001; 14d: *P* = 0.0009. L5 vs. L3, 10 min: *P* < 0.0001; 1 h: *P* < 0.0001; 4 h: *P* < 0.0001; 12 h: *P* < 0.0001; 1d: *P* < 0.0001; 3d: *P* < 0.0001; 7d: *P* < 0.0001; 14d: *P* < 0.0001). (**f**) Quantitative measurement of Evans blue extravasation in sham-operated (n = 3/time point) and SNT rats (n = 4/time point). SNT vs Sham: F_(1, 5)_ = 257.0, ***P < 0.001; 10 min: *P* < 0.0001; 1 h: *P* < 0.0001; 4 h: *P* < 0.0001; 1d: *P* < 0.0001; 7d: *P* < 0.0001; 14d: *P* = 0.0004. (**g**) Cutaneous temperature (footpad) in the L4 dermatome of the SNT ipsilateral, contralateral paws and core (rectal) temperature after L5 SNT (n = 5/group). SNT side vs Contralateral side: F_(1, 8)_ = 24.02, **P = 0.0012; 10 min: *P* < 0.0001; 20 min: *P* < 0.0001; 30 min: *P* < 0.0001; 60 min: *P* < 0.0001; 120 min: *P* < 0.0001; 240 min: *P* < 0.0001. (**h**) Cutaneous temperature (footpad) in the L4 dermatome of the ipsilateral and contralateral paws and core (rectal) temperature after application of 2% lidocaine quickly around L5 spinal nerve for 1 min (n = 3/group). Contralateral side vs Lidocaine L5: F_(1, 4)_ = 87.57, *P* = 0.0007; 10 min: *P* = 0.0002; 20 min: *P* < 0.0001; 30 min: *P* < 0.0001; 60 min: *P* < 0.0001. *P < 0.05, **P < 0.01, ***P < 0.001, ^#^*P* < 0.001. (**a**–**d**,**f**–**h**) Two-way mixed model ANOVA with sidak’s multiple comparisons test. Data are expressed as mean ± SEM.

We transected the L5 spinal nerve by quickly withdrawing a pre-implanted loop suture surrounding the L5 spinal nerve in awake rats and conducted behavioral tests immediately after injury. In L5 SNT rats, the duration of spontaneous foot lifting (SFL), an indicator of spontaneous pain, was sharply increased in the ipsilateral hind paw at 10 min after injury, as compared to that in sham-operated rats (Fig. 1a). SFL in L5 SNT rats remained significantly higher than that of the sham-operated group at postoperative day (POD) 1 and POD4. The paw withdrawal threshold (PWT) to mechanical stimulation (von Frey hair) applied to the ipsilateral hind paw was significantly decreased from 10 min to POD14, as compared to that in sham-operated rats (Fig. 1b). In the hot plate test, the paw withdrawal latency (PWL) to thermal stimulation was significantly decreased from 10 min to POD7 after injury (Fig. 1c). The frequency of licking or biting of the ipsilateral hind paw after cold stimulation (acetone) was significantly increased from 10 min after injury to POD14 (Fig. 1d). In sham-operated group, none of these outcome measures was significantly changed from pre-injury baseline or the naive control group (Fig. 1a–d). These findings suggest that pain after axotomy may develop much sooner in awake animals than previously known^13–16^.

The rat hind paw is innervated by L3–L5 spinal nerves from the medial to the lateral side. Further examination showed that the decrease in PWTs measured in L3 and L4 territories was significantly greater than that in L5 territory (Fig. 1e). This finding suggests that the mechanical hypersensitivity after acute L5 SNT was more prominent in the skin territories of uninjured L3–4 spinal nerves than in those of L5 spinal nerve. Evans blue extravasation is a common measure of neurogenic inflammation^17–20^. Compared to pre-injury level (3.44 ± 0.29 μg/g), the concentration of Evans blue was significantly increased at 10 min after SNT (14.04 ± 1.33 μg/g), peaked at 4 h (24 ± 1.38 μg/g), and gradually decreased from POD1 (18.6 ± 1.35 μg/g; 10 min–POD14, Fig. 1f). Evans blue extravasation was also greater in L3 and L4 territories, as compared to that in L5 territory (Supplementary Fig. 1). Evans blue extravasation did not change significantly after sham operation. Local inflammation is often associated with an increase in skin temperature^21–23^. In line with the Evans blue extravasation findings, skin temperature of the ipsilateral hind paw was significantly elevated from 10 min to 240 min after L5 SNT, as compared to temperature on the contralateral side (Fig. 1g). Blocking the afferent inputs from L5 spinal nerve to the spinal cord by local application of lidocaine (2%) also increased skin temperature of the hind paw for approximately an hour after treatment (Fig. 1h). The core body temperature and skin temperature of the contralateral hind paw after L5 SNT were not changed from pre-injury level. These findings suggest that L5 SNT may quickly trigger neurogenic inflammation in the peripheral tissue, and more so in the territory of neighboring intact spinal nerves.

### Spontaneous activity quickly developed in nociceptive neurons of uninjured L4 dorsal root ganglion (DRG) after L5 spinal nerve transection

**Figure 2:**
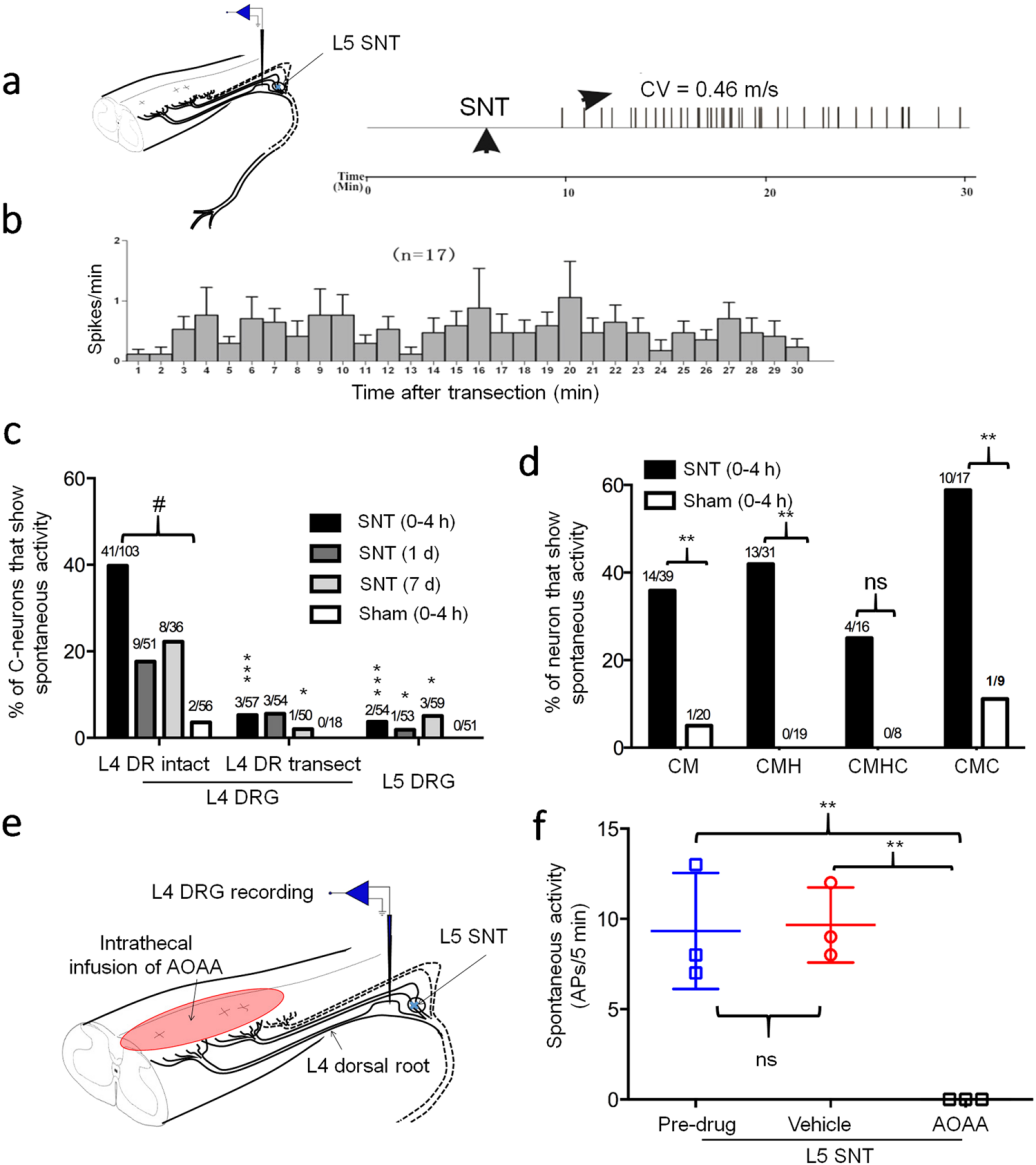
Spontaneous activity in L4 DRG neurons after acute L5 SNT in rats. (**a**) Left: Schematic diagram of the experimental setup. Right: Representative example of an initially quiescent C-nociceptive neuron in L4 DRG that quickly developed spontaneous activity after acute L5 SNT. CV, conduction velocity. (**b**) Mean discharge rates (spikes/min) of C neurons in L4 DRG after acute L5 SNT. (**c**) Left and middle: Percentage of C neurons in L4 DRG that showed SA at different time points after L5 SNT with or without transection of L4 dorsal root. Right: Percentage of C-neurons in L5 DRG that showed SA after L5 SNT without transection of L4 dorsal root. (L4 DR Intact vs L4 DR Transect, 0–4 h: *P* < 0.0001; 1d: *P* = 0.1504; 7d: *P* = 0.0104. L4 DR Intact vs L5 DRG, 0–4 h: *P* < 0.0001; 1d: *P* = 0.0244; 7d: *P* = 0.0405. L4 DR Intact SNT vs Sham, 0–4 h: ^#^*P* < 0.0001, Chi-square test). (**d**) Percent of each subtype of neuron in L4 DRG that showed SA during the first 4 h after L5 SNT or sham operation. (CM: *P* = 0.0099; CMH: *P* = 0.0010; CMC: *P* = 0.0191, Chi-square test). (**e**) Schematic diagram of the recording setup and intrathecal infusion of aminooxyacetic acid (AOAA) at lumbar spinal cord. (**f**) SA of C-nociceptive neurons in L4 DRG after acute L5 SNT followed by intrathecal infusion of vehicle or AOAA (10 μl, 100 mmol, n = 3). One-way ANOVA, F _(1.007, 2.013)_ = 770.5, ***P = 0.0013; Dunnett’s multiple comparisons test, Pre-drug vs after AOAA: *P* = 0.0026; Vehicle vs after AOAA: *P* = 0.0011. *P < 0.05, **P < 0.01, ***P < 0.001, ^#^*P* < 0.001, ns, not significant. Data are expressed as mean ± SEM. CM, C-mechano-sensitive; CMH, C-mechano-heat-sensitive; CMHC, C-mechano-heat-cold-sensitive; CMC, C-mechano-cold-sensitive; APs, action potentials.

We next examined the neurophysiologic mechanisms that may underlie the quick onset of pain after L5 SNT (dissecting scissors cut). Recent microneurography studies in patients suggested that development of SA and ectopic discharge in a subpopulation of DRG neurons after nerve injury might underlie spontaneous pain^1,2^. By conducting highly sensitive *ex vivo* electrophysiology recordings of DRG neurons, we found that 14 nociceptive neurons (C neurons) out of 17 neurons recorded from uninjured L4 DRG in 17 rats developed SA within a few minutes after L5 SNT, and remained active for at least 30 min (Fig. 2a,b). The average rates of SA from all neurons (i.e., neurons with and without SA after SNT) after SNT were shown in Fig. 2a.

DRG neurons were classified based on axon conduction velocity and response properties. The percentage of C neurons in L4 DRG that showed SA within 4 h after L5 SNT was significantly high-er than that after sham operation (Fig. 2c). In contrast, only a few C neurons in L5 DRG showed SA after transection (Fig. 2c). The injury discharge of C neurons in L5 DRG lasted for no more than a minute in our electrophysiological recording(Supplementary Fig. 2), which is in line with findings in a previous study^24^. Based on the response properties, we separated C neurons into different subgroups: C-mechano-sensitive (CM), C-mechano-heat-sensitive (CMH), C-mechano-heat-cold-sensitive (CMHC), and C-mechano-cold-sensitive neurons (CMC). The percentages of CM (14/39, 36%), CMH (13/31, 42%), and CMC (10/17, 59%) neurons in L4 DRG that showed SA at 0–4 h after L5 SNT were significantly higher than that after sham operation (Fig. 2d).

We then examined the source of signals that trigger SA in L4 DRG neurons after L5 SNT. We transected the L4 dorsal root at 10 min before L5 SNT to prevent traveling of retrograde signals from the spinal cord to L4 DRG. Indeed, L4 dorsal root transection significantly reduced the SA in L4 DRG neurons after L5 SNT (Fig. 2c). In a separate experiment, we recorded SA for 5 min after L5 SNT and then intrathecally infused vehicle (artificial cerebrospinal fluid) and aminooxyacetic acid (AOAA, 10 mM, 10 μl), and agar was used to block drug diffusion into DRG bath. AOAA inhibited aminobutyrate aminotransferase activity and increased the level of gamma-aminobutyric acid (GABA)^25^. AOAA significantly reduced SA in L4 DRG neurons, as compared to pre-drug level and that after vehicle treatment (Fig. 2e,f). Together, these findings may suggest that retrograde signals from the spinal cord may elicit SA in L4 DRG neurons after acute L5 SNT.

**Figure 3:**
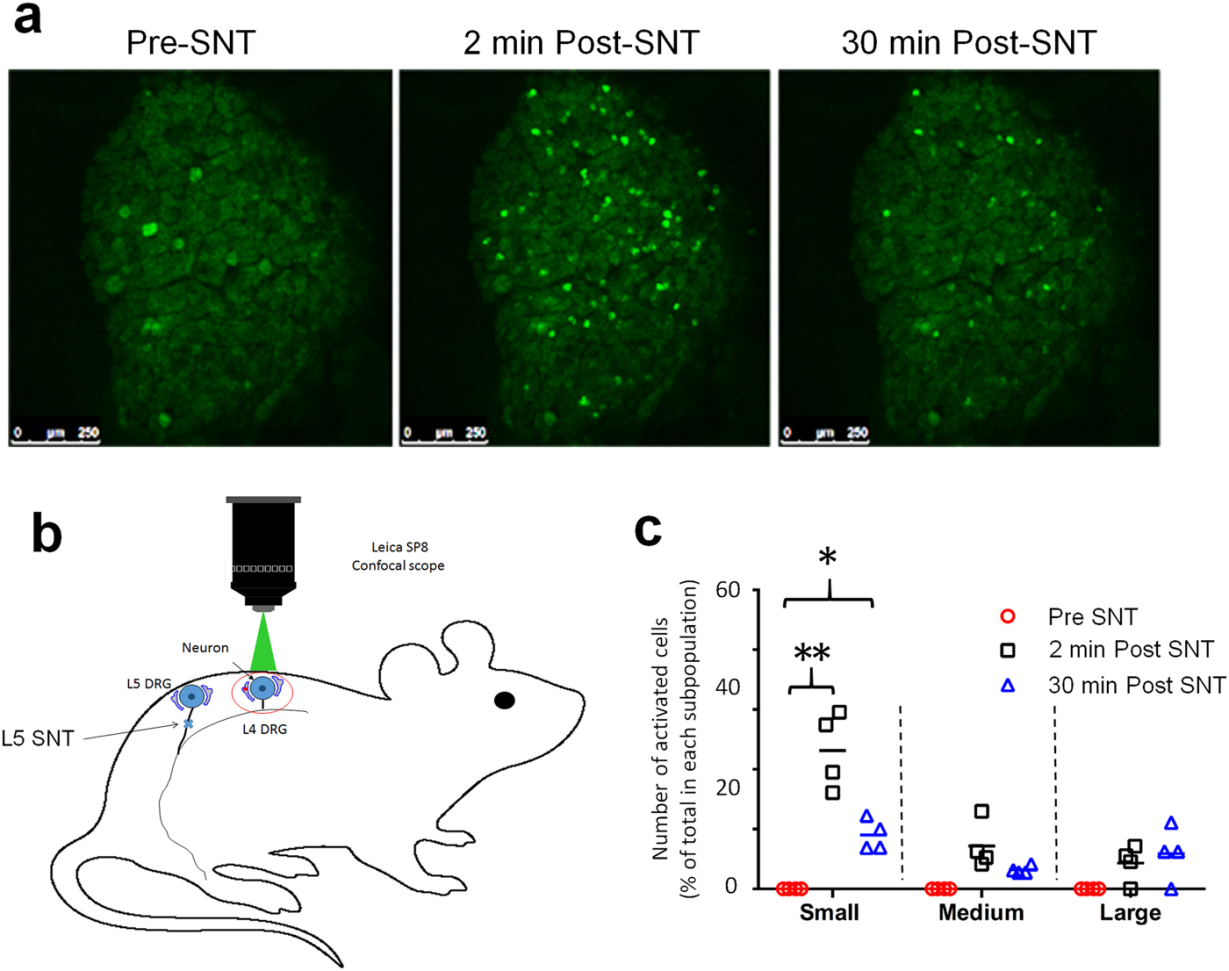
Calcium imaging of activity in L4 DRG neurons after acute L5 SNT in pirt-GCaMP6s mice. (**a**) Representative images of spontaneous activity (SA) in L4 DRG before and at 2 and 30 min after L5 SNT in pirt-GCaMP6s mice. (**b**) Setup for *in vivo* calcium imaging of DRG neurons in pirt-GCaMP6 mice. (**c**) Quantification of number of L4 DRG neurons that showed SA before and 0–30 min after L5 SNT (n = 4). DRG neurons were categorized into three sized-based subgroups with somal areas of < 450 μm^2^ (small), 450–700 μm^2^ (medium), and >700 μm^2^ (large). Two-way ANOVA with Tukey’s multiple comparisons test, F_(2, 6)_ = 98.54, ***P < 0.0001. Pre-SNT vs 2 min Post-SNT, *P* < 0.0001; Pre-SNT vs 30 min Post-SNT, *P* = 0.0118. *P < 0.05, ***P < 0.001. Data are expressed as mean ± SEM.

Using electrophysiologic recording to identify DRG neurons with SA is challenging because it requires sampling a large population of cells. Therefore, we used pirt-GCaMP6s mice to conduct high-throughput calcium imaging of DRG neurons *in vivo*^26–28^. GCaMP is a genetically encoded calcium indicator, and the intensity of its green fluorescence increases robustly when the cell is active^28,29^. In line with electrophysiologic findings, the percentage of small-diameter neurons (<450 μm^2^) in L4 DRG that developed SA was significantly increased at 2 min (139/291 cells in n = 4 mice) and 30 min (47/271 cells in n = 4 mice) after L5 SNT, as compared to that at baseline (0/288 cells, Fig. 3). Thus, both electrophysiologic recording and GCaMP imaging studies suggested a rapid onset of SA in nociceptive neurons from uninjured L4 DRG after L5 SNT.

### Sensitization of nociceptive neurons in L4 DRG after acute L5 spinal nerve transection

**Figure 4:**
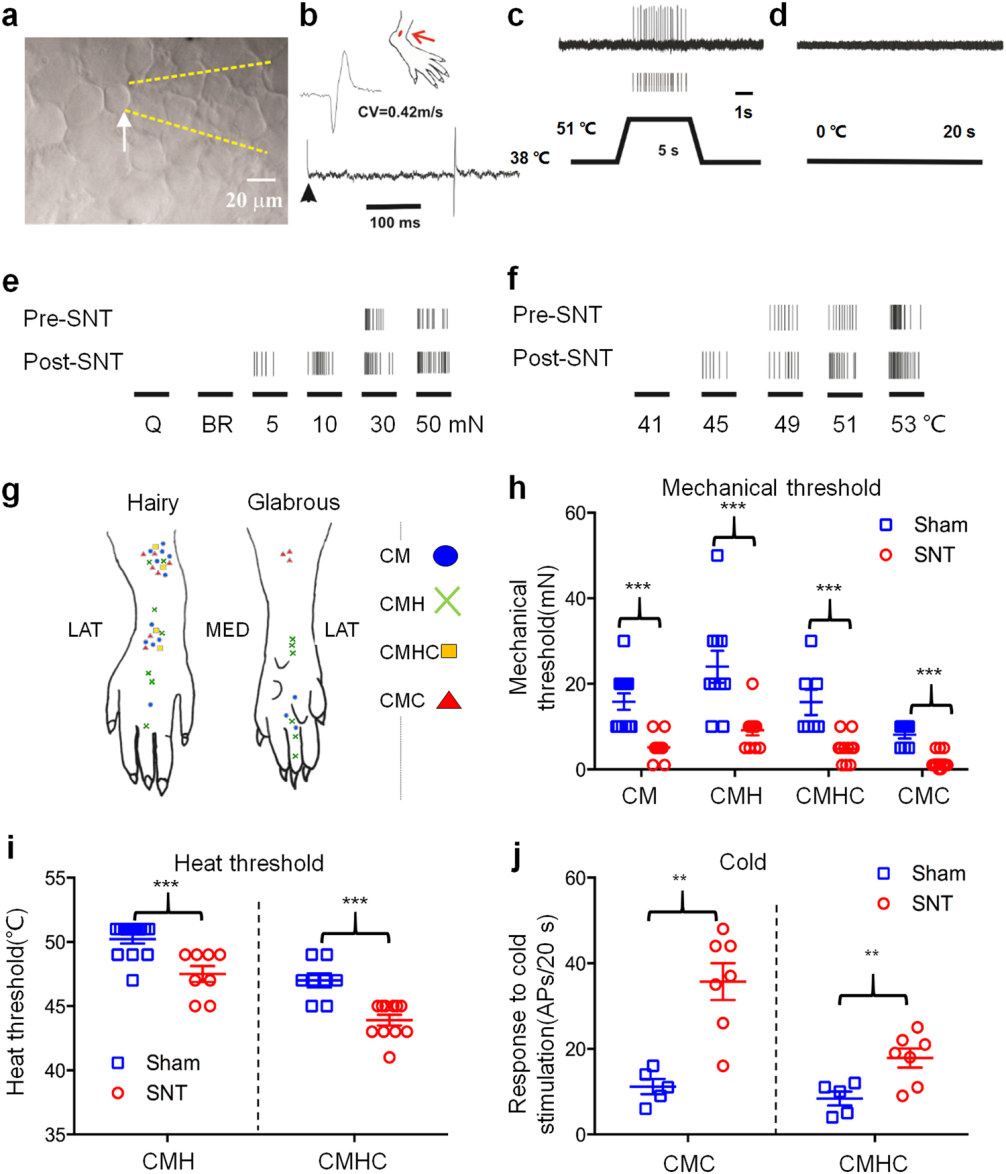
Changes in the excitability of L4 DRG neurons after acute L5 SNT. (**a**) Bright-field image of L4 DRG showing a small-diameter neuron (arrow) and an extracellular electrophysiology recording electrode (dashed yellow lines). (**b**) Conduction velocity (CV; 0.42 m/s) was measured by electrically stimulating the peripheral receptive field (RF, red arrow). (**c**) Action potentials evoked by heat stimulation (51 °C, 5 s) were recorded from the neuronal soma. (**d**) This neuron did not respond to cold stimulus (0 °C, 20 s) and hence was classified as C-mechano-heat-sensitive (CMH). (**e**) Responses of a CMH neuron in L4 DRG to different mechanical stimuli, including a cotton-tipped swab, light brush, and von Frey filaments of multiple bending forces (5, 10, 30, and 50 mN) before and after L5 SNT. (**f**) Responses of a CMH neuron in the L4 DRG to different thermal stimuli (41–53 °C) before and after L5 SNT. (**g**) Schematic diagram of RFs of C neurons that showed spontaneous activity after SNT. LAT: lateral, MED: medial. (**h**) Mechanical thresholds of different subtypes of C neurons in L4 DRG from sham-operated (n = 7–12) and SNT groups (n = 11–13). CM: t_(21)_ = 4.884, *P* < 0.001, two-tailed unpaired t-test; CMC: t_(19)_ = 6.538, *P* < 0.001, two-tailed unpaired t-test; CMH: *P* < 0.001, Mann-Whitney test; CMHC: *P* < 0.001, Mann-Whitney test. (**i**) Heat thresholds of CMH (sham: n = 13; SNT: n = 8) and CMHC (sham: n = 8; SNT: n = 11) neurons. CMH: *P* < 0.001, Mann-Whitney test; CMHC: t_(17)_ = 4.642, *P* < 0.001, two-tailed unpaired t-test. (**j**) Responses of CMC and CMHC neurons to cold stimuli (0 °C, 20 s) in sham-operated (n = 5) and SNT (n = 7) rats. CMC: t_(10)_ = 4.578, *P* = 0.001; CMHC: t_(10)_ = 3.179, *P* = 0.0098; two-tailed unpaired t-test. **P < 0.01, ***P < 0.001, SNT vs. sham. Data are expressed as mean ± SEM.

We further examined whether L5 SNT also increases the evoked response of L4 DRG neurons to mechanical, thermal, and cold stimulation. Stimulus-evoked action potentials (APs) were recorded *ex vivo* from C neurons in L4 DRG for 4 h after L5 SNT or sham operation in rats (Fig. 4a–d). Graded mechanical and heat stimulation evoked more APs in L4 DRG neurons after L5 SNT, as compared to that before injury (Fig. 4e,f). C neurons were characterized and separated into different subtypes (CM, CMH, CMHC, CMC) based on conduction velocity and response properties to mechanical, thermal, and cold stimulation applied to the skin receptive fields (Fig. 4b–d,g). The activation thresholds to mechanical stimulation were significantly decreased in CM, CMH, and CMHC neurons after SNT, as compared to that after sham operation (Fig. 4h). In addition, the number of APs elicited by mechanical stimulation increased significantly after L5 SNT in each subgroup of C neurons (Supplementary Fig. 3). The heat thresholds of CMH and CMHC neurons were significantly lower after L5 SNT than after sham operation (Fig. 4i). In CMH and CMHC neurons, the numbers of APs elicited by heat (45–53 °C, Supplementary Fig. 4) and cold stimuli (0 °C, 20 s, Fig. 4j) were significantly greater in SNT rats than in sham-operated rats.

We also conducted *in vivo* calcium imaging to examine responses of L4 DRG neurons to mechanical and heat stimulation in pirt-GCaMP6s mice. To recruit mechanical sensitive neurons, we used a rodent pincher analgesia meter to stimulate a large area of the hind paw, instead of using von Frey filaments. More small-diameter neurons in L4 DRG were activated by mechanical stimulation (Supplementary Fig. 5a) and heat stimulation (Supplementary Fig. 5b) at 30–60 min after L5 SNT than before injury. Together, these findings suggest a sensitization of primary nociceptive neurons in uninjured L4 DRG soon after L5 SNT, which may correlate with the development of behavioral mechanical and heat hypersensitivities.

### Electrical stimulation of low-threshold afferent fibers reduced spontaneous activity in L4 DRG neurons and attenuated pain behavior after L5 spinal nerve transection

**Figure 5:**
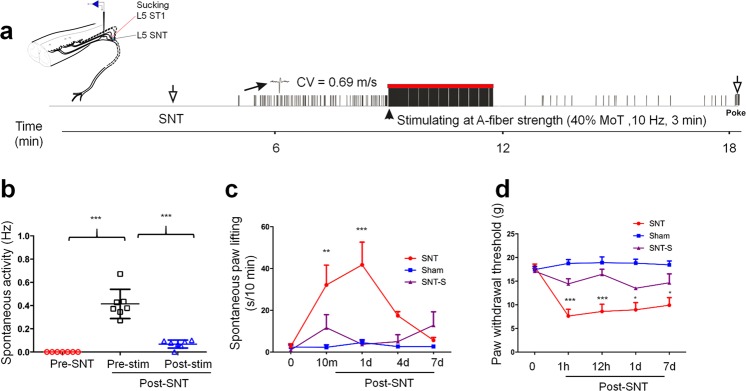
Effects of electrical stimulation of A-fibers on spontaneous activity of L4 DRG neurons and neuropathic pain after acute L5 SNT. (**a**) Left: Schematic diagram of electrical stimulation (S1) applied to the proximal end of transected L5 spinal nerve. Right: An example of spontaneous activity (SA) that quickly developed in an initially quiescent nociceptive neuron (conduction velocity = 0.69 m/s) in L4 DRG after L5 SNT. The SA was attenuated by electrical stimulation of the injured L5 spinal nerve at a low-intensity that activated low-threshold A-fibers (0.5 mA, 10 Hz, 3 min). This neuron responded to mechanical stimulation (poke) at the end of the experiment. (**b**) Quantification of SA in L4 DRG neurons before injury, after SNT, and after electrical stimulation of the L5 spinal nerve (n = 7). One-way repeated measures ANOVA, F_(2, 27)_ = 60.68, ***P < 0.0001; Tukey’s multiple comparisons test, Pre-stim vs Pre-SNT: *P* < 0.0001; Pre-stim vs Post-stim: *P* < 0.0001. (**c**) The increased spontaneous foot lifting after L5 SNT was significantly decreased by A-fiber strength electrical stimulation (n = 6–8/group). F_(2, 19)_ = 13.58, ***P = 0.0002; SNT vs SNT-S: 10 min: *P* = 0.0075; 1d: *P* < 0.0001. (**d**) Changes in the ipsilateral paw withdrawal threshold after sham operation (n = 8), and that after L5 SNT with (SNT-S, n = 6) and without (SNT, n = 8) electrical stimulation. F_(2, 95)_ = 61.89, ***P < 0.0001; SNT vs SNT-S: 1 h: *P* = 0.0003; 12 h: *P* < 0.0001; 1d: *P* = 0.0199; 7d: *P* = 0.0147. *P < 0.05, **P < 0.01, ***P < 0.001. c,d: Two-way mixed model ANOVA with Tukey’s multiple comparisons test. Data are expressed as mean ± SEM.

We determined whether stimulation of low-threshold afferents inhibits SA of C neurons in L4 DRG after L5 SNT. Electrical stimulation was applied via a suction electrode to the remaining L5 spinal nerve proximal to the transection site (Fig. 5a). The stimulation was applied for 10 min after L5 SNT at a low intensity that primarily activates non-nociceptive afferent fibers [Aα/β-fiber, 40% motor threshold (MoT), 10 Hz, 3 min]. The SA frequency in C neurons of L4 DRG at 0–5 min after electrical stimulation (0.02 ± 0.01 Hz) was significantly lower than that at pre-stimulation level (0.3 ± 0.13 Hz, Fig. 5b). Mechanical test stimulation was applied to the skin receptive field of the neurons at the end of the experiment to confirm responsiveness.

Lastly, we examined whether electrical stimulation of low-threshold afferent fibers may also attenuate neuropathic pain-related behavior after L5 SNT. A pair of silver electrodes was pre-implanted under the L5 spinal nerve at 2 days before L5 SNT. Low-intensity electrical stimulation (40% MoT, 10 Hz, 3 min) was applied at the L5 spinal nerve proximal to the transection site. The electrical stimulation was delivered at 1, 6, and 12 h after L5 SNT and then twice daily from POD1 to POD7. Behavioral tests were conducted immediately after each stimulation. The duration of SFL in the ipsilateral hind paw at 10 min and 1 day after L5 SNT was significantly decreased in rats that received electrical stimulation than in those without stimulation (Fig. 5c). The decrease of PWT to mechanical stimulation after L5 SNT also was attenuated by electrical stimulation (Fig. 5d).

## Discussion

Studies of neuropathic pain in animal models have been conducted primarily at hours to days after nerve injury^11,12,30^. Yet, severe pain after nerve injury has been reported in patients much earlier than that observed in animal models^2,4,5,17,31,32^. Because it takes hours for animals to fully recover from general anesthesia after surgery, rapid onset of neuropathic pain-related behavior may not be readily observed^10,12,13,15,16^. In order to avoid these potential confounding factors, we tested a rat model of axotomy induced by acute transection of L5 spinal nerve in awake rats. Using this model, we demonstrated a rapid onset of neuropathic pain-related behavior that included spontaneous pain and hypersensitivity to mechanical, heat, and cold stimuli, and exposed early changes in primary sensory neuronal after acute peripheral nerve injury. The mechanisms underlying the pain after axotomy remain unclear. Previous studies suggested that inflammation in nerve tissue is important to the development of neuropathic pain^10–12^. Yet, changes in gene expression, inflammation, and Wallerian degeneration often develop slowly after injury^11,33^ and may not account for the rapid onset of post-axotomy pain. Our electrophysiology and GCaMP imaging studies revealed increases in SA and responsiveness of nociceptive neurons in neighboring uninjured L4 DRG only a few minutes after L5 SNT. Although the firing rate of individual C neurons was relatively low (<10 APs/5 min), this SA may induce pain owing to the large number of neurons involved.

Compared to previous studies which were conducted at a later time point after spinal nerve ligation (SNL)^7,9^, we observed a higher incidence of C neurons and a lower incidence of A neurons in L4 DRG that show SA soon after L5 SNT. However, the average rates of SA in C neurons were lower than that in the previous studies. Differences in animal models (e.g., SNT versus SNL), post-injury time points, and experimental conditions may partially account for the discrepancies between current observation and previous findings^7,9^. Nevertheless, our finding is in line with the observation that low-frequency activity in C neurons elicits hyperalgesia in humans and rats^18,19^. The hyperexcitability of C neurons in L4 DRG developed quickly after L5 SNT, almost simultaneously with the onset of spontaneous pain and evoked pain hypersensitivity. Both mechanical hypersensitivity and neurogenic inflammation, as indicated by increased Evans blue extravasation, were more prominent in the dermatomes of uninjured spinal nerve. These findings suggest that hyperexcitability of uninjured C neurons may induce neurogenic inflammation and pain hypersensitivity after axotomy.

The spontaneous pain and corresponding electrophysiologic changes witnessed in this study occurred almost immediately after L5 SNT and persisted until pain had transitioned into the chronic phase. The mechanisms that lead to a quick onset of C neuron sensitization in L4 DRG after L5 SNT remain to be determined in a future study. Intriguingly, transection of L4 dorsal root reduced C neuron hyperexcitability in L4 DRG, suggesting that retrograde signals from the spinal cord are important to the sensitization of uninjured DRG neurons after axotomy. Consistent with this notion, intrathecal infusion of AOAA, which would increase the inhibitory tone from GABAergic neurons, induced a similar inhibitory effect. The gate control theory of pain, proposed by Melzack and Wall^20^, postulates that activities of non-nociceptive afferent neurons drive a feed-forward activation of spinal inhibitory neurons to close the “gate” and inhibit spinal nociceptive transmission. However, it is unknown whether A-fiber inputs exert tonic inhibition of primary nociceptive neurons under physiologic conditions. A recent study showed that abolishing low-threshold afferent inputs by demyelinating A-fibers with cobra venom induced a quick onset of heat pain hypersensitivity and an increase of C neuron excitability^21^. Together, these findings suggest that a loss of tonic A-fiber inputs, such as by axotomy or demyelination, might deactivate certain inhibitory interneurons and “open” the gate in the spinal cord dorsal horn, and quickly induce pain. In contrast, low-intensity electrical stimulation at the venom injection site, which primarily activated A-fibers, inhibited SA in C neurons^21^. Here, electrical stimulation of L5 spinal nerve at an intensity that activates low-threshold afferent fibers also attenuated SA of uninjured L4 DRG neurons *in vivo*, and alleviated pain in awake rats after L5 SNT. Our results indicate that disinhibition of C-neurons by the loss of A-fibers input might induce nociceptive activation and pain immediately after nerve injury. These effects could be reversed by compensatory A-fiber inputs via peripheral nerve stimulation. Dorsal root reflex might be involved in the triggering of neurogenic inflammation after nerve injury as observed in this study. The detailed spinal cord neural circuit mechanisms will be further investigated in future studies.

Although some widely adopted preclinical pain models such as severe ongoing pain after intra-plantar injection of formalin and capsaicin^22,23^ were also performed in awake animals, it remains possible that L5 SNT produces more severe, traumatizing and lasting pain to awake animals than other models. In addition, current findings suggest that anesthesia did not impair the rapid increase of neuron excitability in L4 DRG, a possible neurophysiological correlate of the early neuropathic pain after acute L5 SNT. Accordingly, we suggest that further investigation of behavior change and peripheral neuronal mechanisms of pain after acute nerve injury should be conducted with effective anesthesia during surgery.

In summary, our findings show for the first time that peripheral axotomy in rats induced a quick onset of neuropathic pain-related behavior and neurogenic inflammation, which may be associated with increased excitability of adjacent intact primary nociceptive neurons. Current findings may provide the biological basis for developing novel therapeutic strategies for neuropathic pain, including, but not limited to, the pain that develops abruptly after nerve injury.

## Methods

### Animals

Adult female Sprague rats Beijing, China, 128 rats for behavior test; 56 rats for Evans blue test. 16 rats for Skin temperature test; 89 rats for DRG recording, some behavior rats used for recording) and adult Pirt-Cre;Rosa26-flox-stop-flox-GCaMP6s heterozygous mice (25–30 g, both sexes) were used in this study. The animal behavior and electrophysiology studies were conducted at the Peking Union Medical College, China, and was approved by the Institutional Animal Care and Use Committees of the Chinese Academy of Medical Sciences and Institute of Basic Medical Sciences (Project #211-2014). The GCaMP imaging study was conducted at the Johns Hopkins University, USA, and was approved by the Institutional Animal Care and Use Committee. Confirming that all experiments were performed in accordance with relevant guidelines and regulations.

### Behavioral tests

In behavioral experiments, the L5 spinal nerve was separated and encircled loosely with 5-0 silk sutures 1 week before transection. Rats were anesthetized with pentobarbital sodium (50 mg/kg, administered intraperitoneally [IP], Sigma-Aldrich Corp., St. Louis, MO, USA). Under aseptic conditions, the right L5 transverse process was removed, and the L5 and L4 spinal nerves were identified. A suture loop was placed around the L5 spinal nerve and went through a plastic tube with both ends close to the skin incision on the back. For sham-operated rats, the suture loop was just placed adjacent to (but not surrounding) the L5 spinal nerve. The suture was located approximately 3–4 mm proximal to the junction with the L4 nerve. The incision was then closed in layers. The suture ends were placed under the skin when the incision was closed. L5 spinal nerve was transected in awake rats by quickly pulling out the pre-implanted both ends of the suture surrounding the nerve while holding the plastic tube inside the body, so that the suture loop was completely pulled out after cutting through the nerve trunk. After behavior test, we checked L5 spinal nerve all clean cut.

To apply electrical stimulation at the L5 spinal nerve in animal behavioral studies, we implanted a pair of silver electrodes under the L5 spinal nerve proximal to the transection site 1 week before the L5 SNT. The electrodes were custom made with 0.2 mm diameter sliver wires (Cat#782000, A-M systems, Sequim, WA, USA) and wired underneath the skin to a small electric port sutured on the back of rat. The electrical stimuli were generated by a stimulator (SEN-7103, Nihon Kohden, Tokyo, Japan) and isolator (SS-102J, Nihon Kohden), which was connected to the port on the back of each rat before the stimulation. The intensity of electrical stimulation (40% motor threshold (MoT), 10 Hz, 3 min) was pre-tested so that only low-threshold afferent fibers were activated^34,35^.

Rats were acclimatized to the behavioral testing box for 3 consecutive days prior to baseline testing and for 30 min before each test. Acute behavior test acclimatized less than 10 min for the first time point. All behavior test is separately independence test. Every behavior animal to sham VS SNT is 8 VS 8. Mechanical hypersensitivity was examined before surgery and at 10 min, 1 h, 4 h, 12 h, 1 day, 7 days, and 14 days after surgery. Heat and cold hypersensitivity and spontaneous pain were tested before surgery and at 10 min and 1, 7, and 14 days after surgery. The electrical stimuli were delivered at 1, 6, and 12 h after L5 SNT and then twice daily from POD1 to POD7 in the stimulation group, while no stimuli in the control group. Spontaneous pain, cold, mechanical and heat hypersensitivity were tested immediately after the electrical stimulation at 1, 6, and 12 h after L5 SNT and then in POD1, 3, 5, and 7.

#### Mechanical hypersensitivity

Rats were placed in individual acrylic glass boxes with a wire grid bottom. Then, a calibrated electronic von Frey filament (Electronic von Frey 2390-5 Anesthesiometer; IITC Life Science, Woodland Hills, CA, USA) was applied perpendicularly to the plantar surface of the hind paws and held for approximately 3 s. Abrupt paw withdrawal, licking of the paw, or shaking of the paw indicated positive responses. Three measurements were made per side and the average calculated to yield the Paw withdrawal threshold (PWT) in response to mechanical stimulation.

#### Heat hyperalgesia

Thermal hyperalgesia was measured by methods previously described^13,15^. Rats were placed in the acrylic glass box of a thermal testing apparatus (BME-410C Full-Automatic Plantar Analgesia Tester; Institute of Biomedical Engineering, Tianjin, China) and allowed to acclimatize to the apparatus for another 30 min. A movable radiant heat source located under the glass floor was focused onto the plantar surface of the hind paw (51 °C). The maximum automatic cutoff time was set at 20 s to prevent potential tissue damage. Abrupt paw withdrawal, paw licking, or paw shaking indicated positive responses. Three measurements were made per side, and an average of the readings was calculated to yield the paw withdrawal latency to heat stimulation.

#### Cold allodynia

After rats were acclimatized, 0.1 ml of acetone was gently applied to the plantar surface of the hind paw. Rapid withdrawal of the hind paw, paw licking, and paw shaking in response to the spread of the acetone over the planter surface of the hind paw were considered positive responses. Three 3-min tests were conducted on each hind paw at 3-min intervals. An increase in the number of positive responses was interpreted as the development of increased cold sensitivity^36,37^.

#### Spontaneous pain

The duration of spontaneous foot lifting (SFL) was used as an indication of spontaneous pain after nerve injury. It was measured as the cumulative duration (in seconds) per 10 min in which rats lifted their ipsilateral hind paw, often accompanied by shaking or licking. Foot lifting associated with exploratory behavior, locomotion, body repositioning, and grooming was excluded^6,38^. An increase in the duration of SFL compared with the sham-operated group was interpreted as the development of spontaneous pain^13,37^.

### Electrophysiologic recordings

A surgical procedure similar to that used in the rat behavior, but the L5 spinal nerve was separated and transected with scissors and rats were anesthetized with sodium pentobarbital. Primary sensory neurons innervating the skin of the hind limb were recorded with an *ex vivo* extracellular electrophysiologic preparation, as previously described^8,9,39,40^. Rats were used for electrophysiologic recording at 0–4 h, 1 day, and 7 days after surgery. A chosen cell body was suctioned into the mouth of a glass micropipette (tip diameter, 20–25 µm) filled with the bath solution, the extracellular artificial spinal fluid solution contained 120 mM NaCl, 3 mM KCl, 1.1 mM CaCl_2_, 10 mM glucose,0.6 mM NaH_2_PO_4_, 0.8 mM MgSO_4_, 18 mMNaHCO_3_ (pH 7.4) with NaOH. APs were recorded extracellularly by using a Multiclamp 700B amplifier and Digidata 1440A. A peripheral receptive field was identified by exploration of the hind limb and application of various handheld stimuli. Applicators included a cotton-tipped swab and camel-hair brush (for innocuous mechanical stimuli), gentle pinching or indentation with a glass probe, and von Frey filament with a fixed tip diameter (200 μm) to deliver different bending forces (for mechanical stimuli), a temperature-controlled chip-resister heating probe for a series of heat stimuli, and ice water. Mechanical stimuli were delivered by a series of von Frey filaments applied to the receptive field for 1 s in ascending order (5, 10, 30, and 50 mN). A series of heat stimuli was applied with a baseline of 38 °C, a rapid temperature ramp, a 5-s plateau of 41, 45, 49, 51, or 53 °C; and then back to baseline. Water ice (0 °C), used as the cold stimulus, was applied to the receptive field as previously described^39–41^.

#### Classification of DRG neurons

Each DRG neuron was classified as C, Aδ, or Aβ by its axonal conduction velocity (<1.3, 1.3~12, or >12 m/s, respectively)^39,41–43^. DRG neurons with hairy and glabrous receptive fields were included. C neurons were further classified into CM, CMH, CMHC, or CMC subgroups if they were responsive to the following stimulation applied to the receptive field: mechanical stimulation (pinch pressure) only, mechanical and noxious thermal stimulation (51 °C, 5 s), mechanical and noxious thermal and cold stimulation (water -ice, 0 °C, 20 s), and mechanical and noxious cold stimulation, respectively.

#### Criteria for defining a spontaneously active neuron

For each neuron identified, a continuous recording was obtained for 3 min without any external stimulation. If spontaneous ongoing discharge occurred during this period, the neuron was classified as spontaneously active. Any “injury discharge” that appeared on occasion immediately after electrode touching and lasted less than 30 s was ignored.

### Evans blue extravasation

A surgical procedure similar to that used in the rat behavior. Rats were anesthetized with sodium pentobarbital, injected with Evans blue (50 mg/kg, dissolved in 1 ml saline, administered intravenously; BDH, UK), and perfused transcardially with 0.1 M phosphate-buffered saline 20 min later. The skin of the hind paw was photographed for qualitative measurement and then removed for quantitative measurement of Evans blue extravasation according to the method described^44^. Briefly, the removed skin was incubated in *N*, *N*-dimethylformamide overnight at 55 °C to allow Evans blue to completely dissolve in the solvent. The following day, the Evans blue absorbance value was measured by a Microplate spectrophotometer at 630 nm. The concentration of Evans blue was normalized to a reference sample of Evans blue (0–9.6 μg/ml).

### Skin temperature measurement

SNT and Lidocaine (2% in saline around the L5 spinal nerve for 1 min) group procedure similar to that used in the rat behavior but rats were anesthetized with sodium pentobarbital. The skin temperature of the hind paw was measured at room temperature (22 ± 0.2 °C) by inserting an electric thermometer (diameter: 1 mm) in between two toes. The value was recorded on a double-channel chart recorder.

### GCaMP imaging

In calcium imaging studies, a procedure similar to that used in the rat electrophysiologic studies was applied to mice but with no electrode implantation. Pirt-GCaMP6s mice were anesthetized with 2% isoflurane, and the lumbar L4 DRG ipsilateral to the nerve injury was exposed as described in our previous study^26,28,45^. During surgery, mice were kept on a heating pad to maintain body temperature at 37 ± 0.5 °C as monitored by a rectal probe. To have a clean image of the sensory cell bodies neurons, the dura mater and the arachnoid membranes were carefully opened and removed using microdissection forceps. *In vivo* calcium imaging of the whole L4 DRG was performed immediately after SNT for 1–6 h as previously described^28^. Mice were laid in the prone position on a custom-designed microscope stage. The spinal column was stabilized with custom-designed clamps to minimize movements caused by breathing and heartbeats. All *in vivo* imaging experiments were performed using a Leica SP8 confocal microscope (Leica). For GCaMP excitation, a laser wavelength at 488 nm (2% laser power) was used, and the images were acquired at a bidirectional scan speed of 600 Hz. Raw image stacks were imported into ImageJ (National Institutes of Health, Bethesda, MD, USA) for further analysis. The train burst width was 8 s. After 16 s of baseline imaging, we applied the test stimulus to the hind paw. For spontaneous activity recording, isoflurane level was lowered to 1% and the DRG was imaged continuously for 40 min with no stimulation.

#### Stimulus delivery

We applied a rodent pincher analgesia meter instead of a von Frey filament as a mechanical stimulus to the ipsilateral hind paw to ensure that most mechanically sensitive neurons were investigated. Because of the large contact surface area of the pincher, the force needed to evoke pain responses was much higher than that normally applied with von Frey filaments. We determined in advance and in our previous study that the PWT of the pincher was 500 g in normal mice^28^. A series of mechanical stimuli was sequentially applied to the hind paw, and the responses of all DRG neurons were recorded. The duration of the pressure was 8 s after 16 s of baseline imaging. Similarly, we applied thermal stimuli (45–51 °C, 5 s) to study responses to thermal stimulation after 16 s of baseline imaging^28^.

#### Imaging data analysis

Raw TIFFs were exported and analyzed with ImageJ as previously described^28^. An experimenter manually traced the activated cells and determined cell size and relative fluorescent intensity off-line after completing the study. Briefly, Small, medium, and large diameter neurons were defined as having somal areas of <450 μm^2^, 450–700 μm^2^, and >700 μm^2^, respectively. The average fluorescence intensity in the baseline period was taken as F0 and measured as the average pixel intensity during the first two frames of each imaging experiment. The maximum fluorescence intensity, Ft, was measured by calculating the average (peak − background) pixel values in a given region of interest for each image frame recorded during a time interval before and during the stimulation period. The Ft was then used to calculate ΔF/F using the formula ΔF/F = (Ft − F0)/F0. We used ImageJ or Fiji (National Institutes of Health) and LIF (Leica Microsystems GmbH) to analyze calcium imaging data using standard functions and a custom macro. An activated neuron to the stimulation was defined by Ft/F0 > 1.2, as that shown in previous studies^26–28^.

### Statistical analysis

Data analysis was performed by using Prism 6.0 statistical program (GraphPad Software, Inc.). Raw data were first evaluated for Gaussian distribution by using the D’Agostino & Pearson test (n > 8) or KS normality test (n < 8). Normally distributed data were analyzed with using parametric statistics (two-way analysis of variance (ANOVA), one-way ANOVA, unpaired two-tailed Student t test, and paired two-tailed Student t test). Data (or after log transform) that did not meet the basic assumptions for parametric testing were analyzed with using nonparametric statistics (Mann–Whitney U and Kruskal–Wallis test). Chi-square test was used to compare differences in the percentage of C neurons that showed SA between different experimental conditions. Data were expressed as mean ± standard error of mean, or as percentages where appropriate. A value of *P* < 0.05 was considered significant.

## Supplementary information

Supplementary Figure 1-5

## Data Availability

The datasets generated during and/or analyzed during the current study are available from the corresponding author on reasonable request.
