# Supplementary material for: Adjacent intact nociceptive neurons drive the acute outburst of pain following peripheral axotomy
Source: Sci Rep. 2019 May 21;9:7651. doi: 10.1038/s41598-019-44172-9 (PMC6529466; doi:10.1038/s41598-019-44172-9)
Supplement: Supplementary file 1 — Supplementary Figure 1-5 [file 41598_2019_44172_MOESM1_ESM.docx]

**SUPPLEMENTARY INFORMATION**

**Adjacent intact nociceptive neurons drive the acute outburst of pain following peripheral axotomy**

Zhiyong Chen^1,2,3†^, Tao Wang^1,4†^, Yehong Fang^1,2^, Dan Luo^5^, Michael Anderson^3^, Qian Huang^3^, Shaoqiu He^3^, Xiaodan Song^3,6^, Huan Cui^1,2^, Xinzhong Dong^7,8,9^, Yikuan Xie^1,2^, Yun Guan^3,8^*, Chao Ma^1,2^*

^1^ Institute of Basic Medical Sciences, Department of Human Anatomy, Histology and Embryology, Neuroscience Center, Chinese Academy of Medical Sciences, School of Basic Medicine, Peking Union Medical College. Beijing, 100005, China.

^2^ Joint Laboratory of Anesthesia and Pain, Peking Union Medical College. Beijing, 100730, China.

^3^ Department of Anesthesiology and Critical Care Medicine, Johns Hopkins University, School of Medicine, Baltimore, Maryland 21205, USA.

^4^ Department of Neurosurgery, Xuanwu Hospital, Capital Medical University, Beijing, 100053, China.

^5^ National Key Laboratory of Medical Molecular Biology & Department of Immunology, Institute of Basic Medical Sciences, Chinese Academy of Medical Sciences, Beijing, 100005, China.

^6^ College of Pharmacy, Harbin Medical University, Harbin, 150081, China.

^7^ The Solomon H. Snyder Department of Neuroscience, Center for Sensory Biology, Johns Hopkins University, School of Medicine, Baltimore, Maryland, 21205, USA.

^8^ Department of Neurological Surgery, Johns Hopkins University, School of Medicine, Baltimore, Maryland 21205, USA.

^9^ Howard Hughes Medical Institute, Johns Hopkins University, School of Medicine, Baltimore, Maryland, 21205, USA.

^†^These authors contribute equally to this work.

*Corresponding authors:

Chao Ma, MD, PhD, Department of Human Anatomy, Histology and Embryology, Institute of Basic Medical Sciences, Neuroscience Center, Chinese Academy of Medical Sciences, School of Basic Medicine, Peking Union Medical College. No. 5 Dongdansantiao, Beijing, 100005, China. Phone/Fax: +86-10-69156469; E-mail: [machao@ibms.cams.cn](mailto:machao@ibms.cams.cn).

Yun Guan, MD, PhD, Division of Pain Medicine, Department of Anesthesiology and Critical Care Medicine, the Johns Hopkins University, 720 Rutland Ave., Ross 350, Baltimore, MD 21205. Phone: 410-502-5510; Fax: 410-614-2109; E-mail: yguan1[@jhmi.edu](mailto:sraja@jhmi.edu).


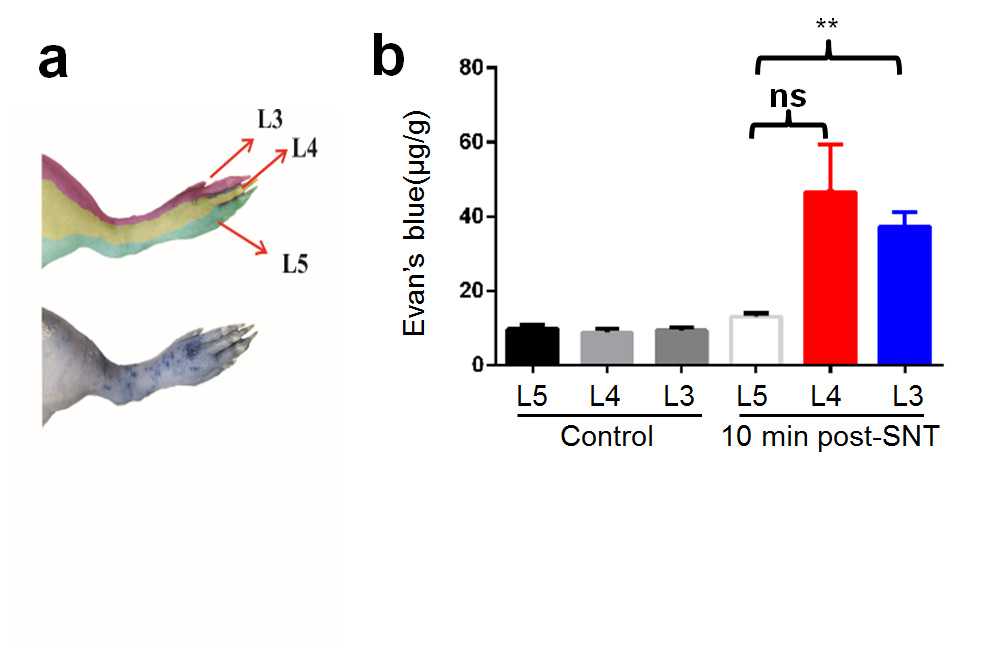
**Supplementary Figure 1**. Evans blue extravasation in skin territories of L3 and L4 spinal nerves after acute L5 SNT. (a) An example image of Evans blue extravasation in the ipsilateral hind paw after an L5 SNT. Territories of L3, L4, and L5 spinal nerves are marked in different colors. (b) Quantification of Evans blue extravasation in territories of L3, L4, and L5 spinal nerves after SNT or sham operation (control; n = 4 rats/group). ***P* < 0.01 vs. L5; ns, not significant. One-way ANOVA. Data are expressed as mean ± SEM.


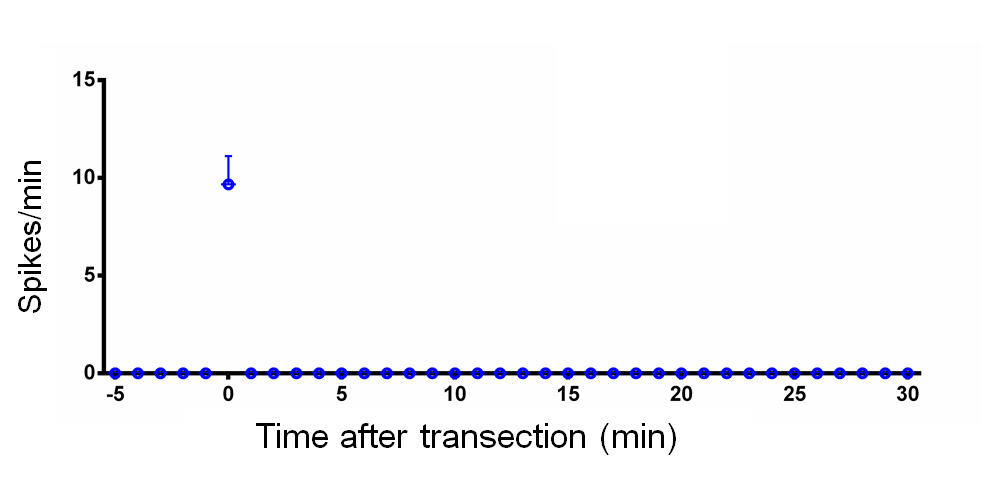


**Supplementary Figure 2**. (a) Mean discharge rates (spikes/min) of C neurons in L5 DRG after acute L5 SNT (n=3).


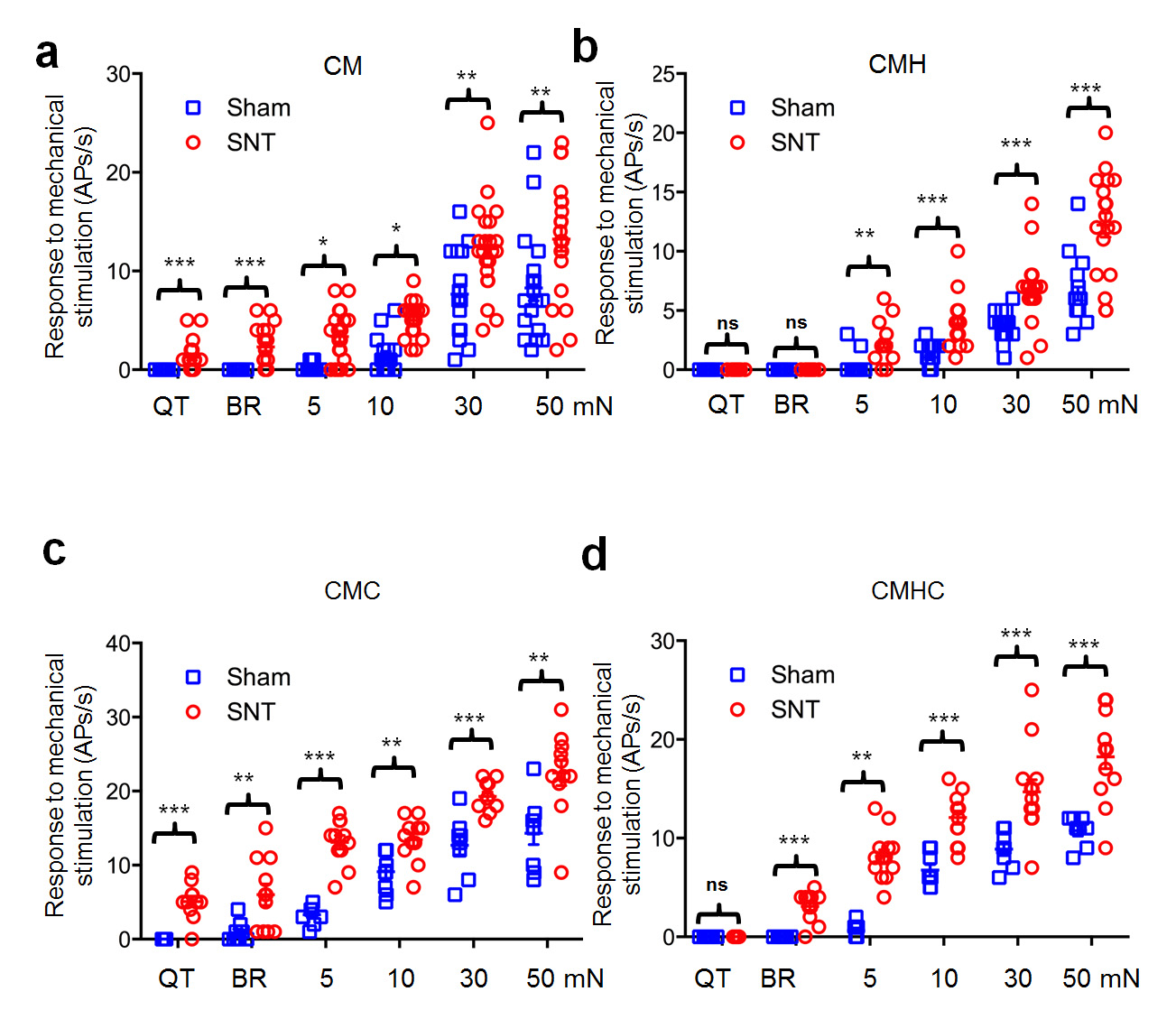


**Supplementary Figure 3**. Activity of different subgroups of C-neurons in L4 DRG evoked by mechanical stimulation after L5 SNT or sham operation in rats. Responses (action potentials [APs]/s) evoked by mechanical stimuli in (a) CM neurons (Sham, n = 18; SNT, n = 22), (b) CMH neurons (Sham, n = 19; SNT, n = 18), (c) CMHC neurons (Sham, n = 8; SNT, n = 13), and (d) CMC neurons (Sham, n = 9; SNT, n = 11) in L4 DRG. **P* < 0.05, ***P* < 0.01, ****P* < 0.001, SNT vs. Sham; ns, not significant. The normality of data distribution was checked by KS normality test. Normally distributed data were analyzed by two-tailed Student’s t-test. Otherwise, data were analyzed by nonparametric tests (Mann-Whitney test). Data are expressed as mean ± SEM.QT: Q-tip. BR: brush.


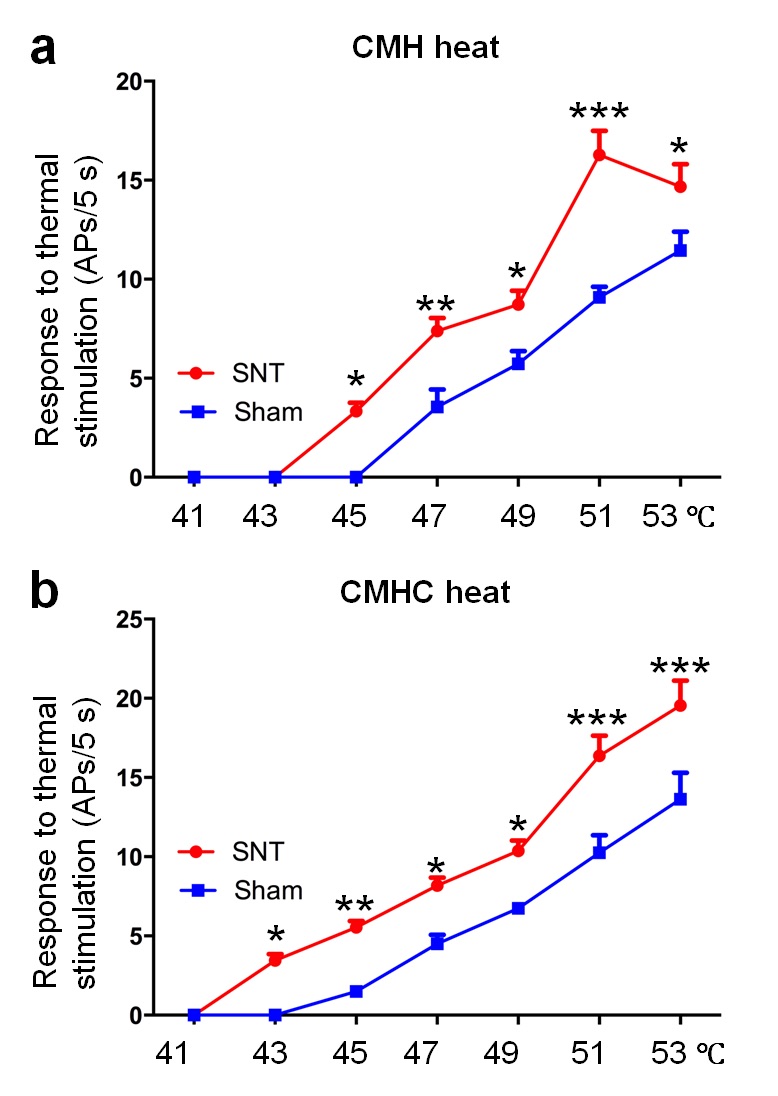


**Supplementary Figure 4.** Activity of different subgroups of C-neurons in L4 DRG evoked by heat stimulation after L5 SNT or sham operation. Responses (action potentials [APs]/5 s) of (a) CMH (Sham, n=11; SNT, n=18), and (b) CMHC neurons (Sham, n=8; SNT, n=11) to heat stimuli (5 s). **P* < 0.05, ***P* < 0.01, ****P* < 0.001, SNT vs. Sham. Two-way ANOVA with Sidak's multiple comparisons test. Data are expressed as mean ± SEM.


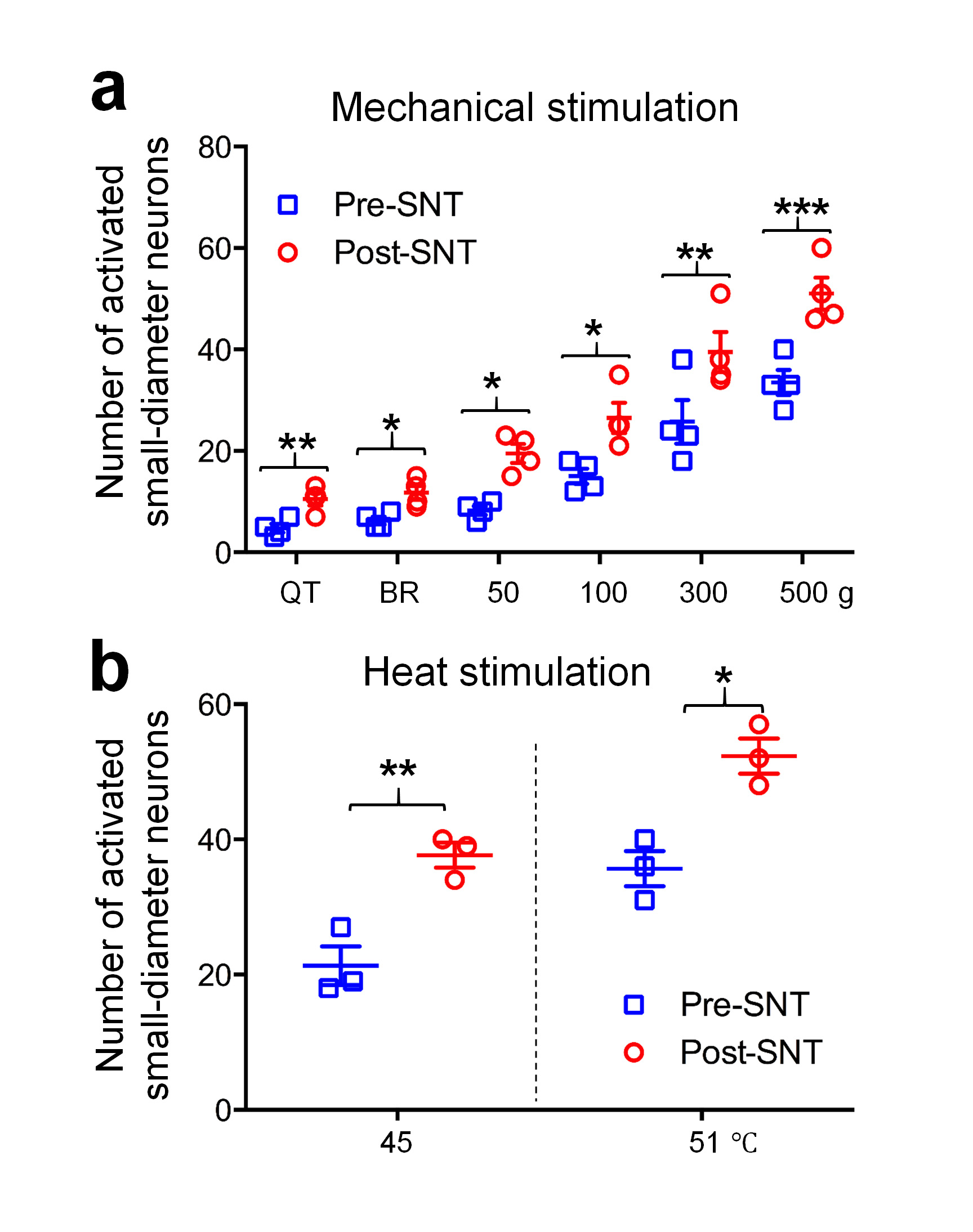


**Supplementary Figure 5**. Calcium imaging of evoked activity in L4 DRG neurons of pirt-GCaMP6 mice after L5 SNT. (a) Average number of neurons in L4 DRG that were activated by mechanical stimuli before and after L5 SNT (n = 4). (b) Average number of neurons in L4 DRG that were activated by heat stimuli (n = 3). **P* < 0.05, ***P* < 0.01, ****P* <0.001, pre-SNT vs. post-SNT. The normality of data distribution was checked by KS normality test. Normally distributed data were analyzed by two-tailed Student’s t-test. Otherwise, data were analyzed by nonparametric tests (Mann-Whitney test). Data are expressed as mean ± SEM. QT: Q-tip. BR: brush.
